# Supplementary material for: Nanometer-precision non-local deformation reconstruction using nanodiamond sensing
Source: Nat Commun. 2019 Jul 22;10:3259. doi: 10.1038/s41467-019-11252-3 (PMC6646314; doi:10.1038/s41467-019-11252-3)
Supplement: Supplementary file 1 — Supplementary Information [file 41467_2019_11252_MOESM1_ESM.pdf]

Supplementary Information

for

Nanometer-precision non-local deformation reconstruction using nanodiamond sensing

Xia et al.

Contents

Supplementary Note 1. Setup 2

Supplementary Note 2. Method of non-local deformation measurement...................................... 4

Supplementary Note 3. Method validation 10

Supplementary Note 4. Reconstructing the deformation of.. 12

Supplementary Note 5. Simulation of the PDMS deformation. 21

Supplementary Note 6. Validation of specific contact models.. 22

Supplementary Note 7. Deformation reconstruction of gelatin upon AFM indentation. 24

Supplementary Note 8. Simulation of the gelatin deformation. 28

Supplementary Note 9. Sensitivity of the rotation measurement of the surface ND.................... 30

Supplementary References... 30

Supplementary Note 1. Setup

We constructed a confocal-atomic force microscopy (AFM) correlation microscope to enable the nonlocal deformation reconstruction via nanodiamond (ND) rotation measurement (Supplementary Fig. 1). The optically detected magnetic resonance (ODMR) measurements were carried out using a home-built laser scanning confocal microscope. A 532 nm laser was adopted (MGL-III-532-200 mW, CNI), and reduction of its power fluctuation down to 0.1% was realized by applying a proportional integral derivative (PID) feedback control. A Nikon 100x (1.45 NA) oil immersion objective lens was used to collect the fluorescence of NDs, which was then detected by an avalanche photodiode (APD, SPCM-AQRH-15-FC, Excelitas) and counted by NIDAQ (PCIe-6363, National Instrument).

A microwave (MW) source (N5171B EXG Signal Generator, Keysight) and an amplifier (ZHL-16W-43-S+, Mini-Circuit) were used to generate microwave for ODMR spectrum acquisition. To achieve high microwave efficiency, the MW was delivered to the NDs by an Ω-shaped microstructure (diameter, 800 μm).

The AFM scanning head (BioScope Resolve, Bruker) was mounted on the confocal microscope to measure the topography and to apply indentation. A shielding was built to isolate the confocal-AFM microscope from external environment using sound-absorbing foams. A temperature PID control module was used to keep the temperature fluctuation < 0.1 °C. This way, the sample drifting was minimized during the indentation experiments.

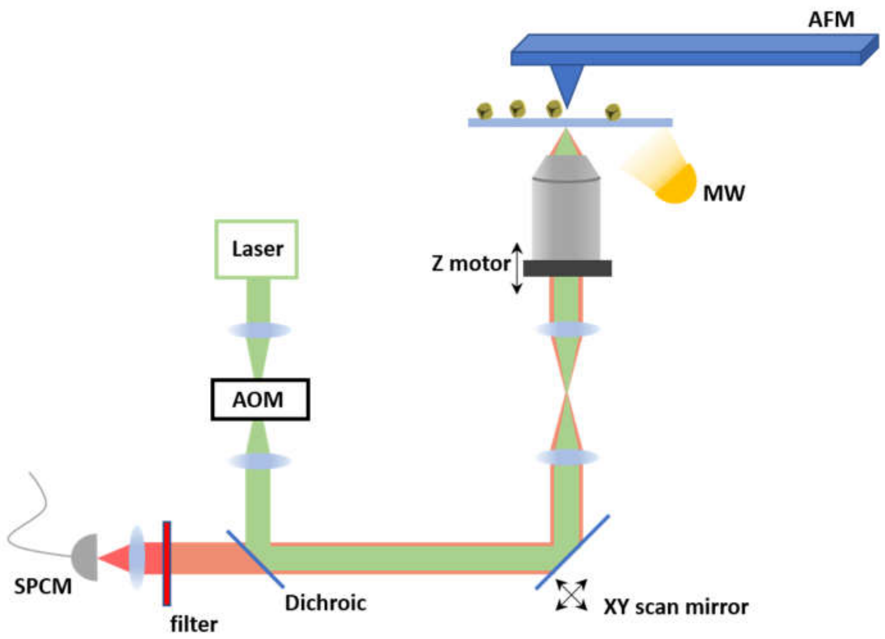

Supplementary Fig. 1 | Schematic of the setup. AOM: acousto-optic modulator, MW: microwave, AFM: atomic force microscopy, SPCM: single photon counting modules.

A peak force tapping mode was applied to obtain the topography information of the samples. Before the indentation experiments, correlating the confocal system and the AFM system was realized in two steps. We first manually overlapped the excitation laser spot with the AFM tip under the top view camera on top of the AFM. Then, an AFM topography image and a fluorescence image were captured and an overlapping between the two was established by comparing the patterns of the NDs distribution in the two images.

In the gelatin indentation experiments, the size (height) of gelatin particles (~10 μm) was much larger than the size of NDs (~ hundreds of nanometers), which made it difficult to resolve the NDs on the gelatin in the AFM image. The correlation between the AFM and the fluorescence images was established by overlapping the fluorescence image and AFM image of NDs on the substrate (cover slide). For the NDs on the gelatin particles, their coordinates were determined

from the fluorescence images instead of the AFM ones. Consequently, the lateral spatial resolution of the gelatin deformation measurements was limited by the optical resolution (~300 nm).

Supplementary Note 2. Method of non-local deformation measurement

(1) ND orientation and rotation

There are four ODMR-inequivalent NV axes in a diamond lattice, (111), $(1\bar{1}\bar{1})$, $(\bar{1}1\bar{1})$ and $(\bar{1}\bar{1}1)$[1], as shown in Supplementary Fig. 2a. The orientation of an ND can be unambiguously defined using the NV axes. We first used the orientation of a certain ND (labelled as ND0) to establish the laboratory coordinates (Supplementary Fig. 2a) by choosing the (111) direction ($\mathbf{NV}_1^0$) as the *z*-axis of and the $(1\bar{1}\bar{1})$ direction ($\mathbf{NV}_2^0$) in the *x*-*z* plane. The four NV orientations $\mathbf{NV}_i^0$ of ND0 can then be written as unit vectors as

$$\mathbf{NV}_1^0 = (0,\ 0,\ 1), \qquad \mathbf{NV}_2^0 = \frac{1}{3}\left(2\sqrt{2},\ 0,\ -1\right),$$
$$\mathbf{NV}_3^0 = \frac{1}{3}\left(-\sqrt{2},\ -\sqrt{6},\ -1\right), \quad \mathbf{NV}_4^0 = \frac{1}{3}\left(-\sqrt{2},\ \sqrt{6},\ -1\right). \tag{1}$$

The orientation of an arbitrary ND (ND-I) is specified by the Euler angles (α, β, γ) (Supplementary Fig. 2b), the angles of three sequential rotations that transfer the orientation of ND0 (whose crystallographic directions set the laboratory coordinate system) to that of ND-I: first a rotation of α about the z-axis in laboratory coordinate system (which transfers the y-axis to $\mathbf{N}(y)$); then a rotation of β about $\mathbf{N}(y)$ (which transfers the z-axis to the z'-axis); and finally a rotation of γ about the z'-axis. The rotation matrix of such an operation is[2]

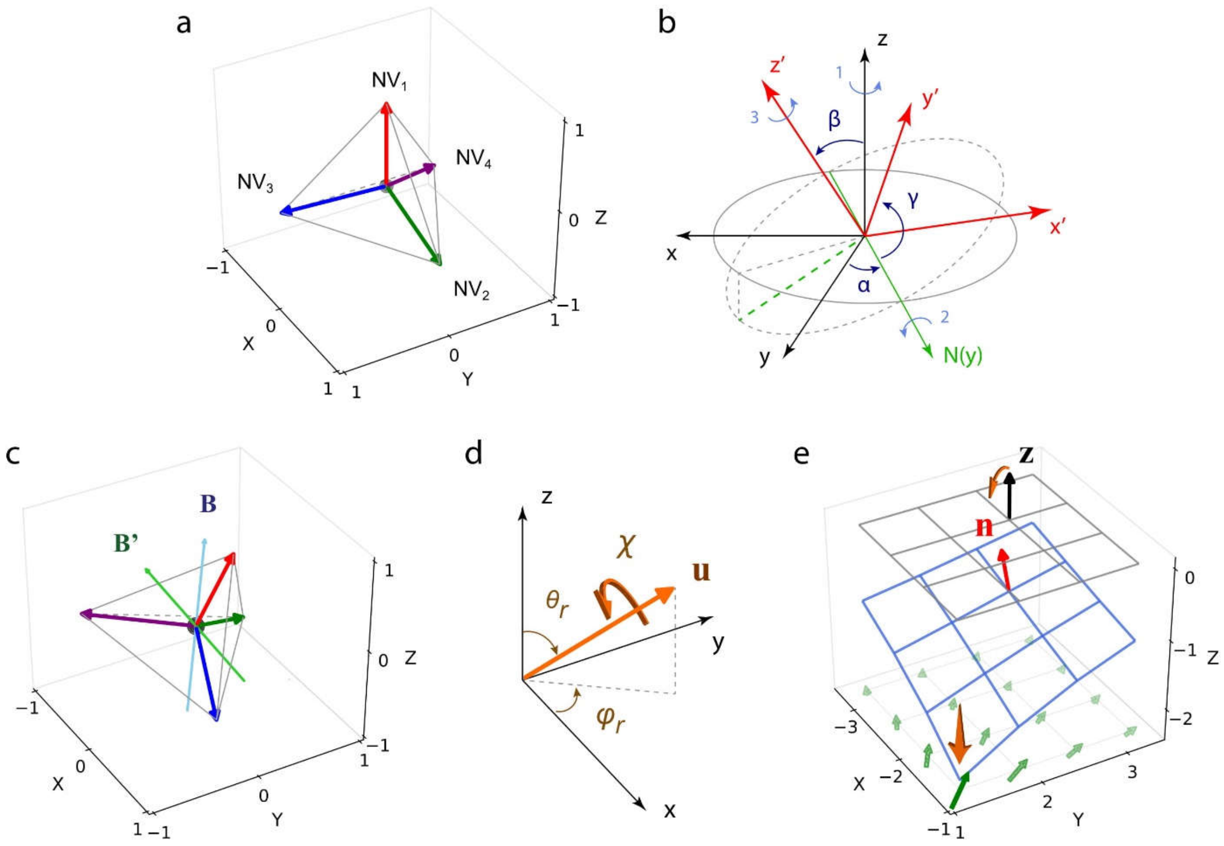

Supplementary Fig. 2 | Geometry of the nanodiamond (ND) rotation measurement. **a,** The four crystallographic orientations of the NV centers in a certain ND (labelled as ND0). The laboratory coordinates are chosen such that (111) direction ($\mathbf{NV}_1^0$) is along the *z*-axis of and the $(1\bar{1}\bar{1})$ direction ($\mathbf{NV}_2^0$) is in the *x*-*z* plane. **b,** The Euler angles representing the orientation of an arbitrary ND, obtained from the orientation of ND0 by three rotations in sequence (1, 2, and 3). **c,** The two magnetic fields and the orientation of the ND used in the simulations. **d,** An rotation with the rotation axis (orange arrow) defined by (θ_r,ϕ_r) and the rotation angle χ. **d,** Illustration of the sample surface with (blue grid) and without (grey grid) indentation. The black and red arrows are the normal vectors of the surfaces. The green arrows are the surface gradient field.

$$\mathbf{R}_\mathrm{I}(\alpha,\beta,\gamma) = \mathbf{R}_z(\alpha)\mathbf{R}_y(\beta)\mathbf{R}_z(\gamma) = \begin{pmatrix} c_\alpha c_\beta c_\gamma - s_\alpha s_\gamma & -c_\gamma s_\alpha - c_\alpha c_\beta s_\gamma & c_\alpha s_\beta \\ c_\alpha s_\gamma + c_\beta c_\gamma s_\alpha & c_\alpha c_\gamma - c_\beta s_\alpha s_\gamma & s_\alpha s_\beta \\ -c_\gamma s_\beta & s_\beta s_\gamma & c_\beta \end{pmatrix}, \qquad (2)$$

where $\mathbf{R}_z$ and $\mathbf{R}_y$ are the matrices of the rotations about the z-, and y-axes, respectively, and $c_x = \cos x$ and $s_x = \sin x$. The corresponding NV orientations in ND-I are related to those of ND0 by $\mathbf{NV}_i^\mathrm{I} = \mathbf{R}_\mathrm{I}(\alpha,\beta,\gamma)\mathbf{NV}_i^0$. Figure S2c shows the orientation of an ND with $(\alpha,\beta,\gamma) = (45°, 20°, 65°)$.

The rotation of an ND can be described by the rotation axis $(\theta_\mathrm{r}, \phi_\mathrm{r})$ (in the spherical coordinates) and the rotation angle χ (see Supplementary Fig. 2d). The rotation matrix **R** is defined by[2]

$$\mathbf{R}(\theta_\mathrm{r}, \phi_\mathrm{r}, \chi)\mathbf{v} = (\cos\chi)\mathbf{v} + (\sin\chi)\mathbf{u}\times\mathbf{v} + (1-\cos\chi)\mathbf{u}\mathbf{u}\cdot\mathbf{v}, \tag{3}$$

where **v** is an arbitrary vector, and $\mathbf{u} = (\sin\theta_\mathrm{r}\cos\phi_\mathrm{r}, \sin\theta_\mathrm{r}\cos\phi_\mathrm{r}, \cos\theta_\mathrm{r})$ is the unit vector of the rotation axis. The orientation of an ND after the rotation is characterized by the Euler angles $(\alpha', \beta', \gamma')$. The rotation $\mathbf{R}(\theta_\mathrm{r}, \phi_\mathrm{r}, \chi)$ of the ND was obtained by comparing its Euler angles before and after the operation, that is $\mathbf{R}(\theta_\mathrm{r}, \phi_\mathrm{r}, \chi)\mathbf{R}_\mathrm{I}(\alpha, \beta, \gamma) = \mathbf{R}_\mathrm{I}(\alpha', \beta', \gamma')$.

(2) Determination of the NV orientations

In the presence of known magnetic field(s) (calibration of the magnetic fields is described in Supplementary Note 3), the orientations of NV centres can be determined from the experimentally measured frequency shift of the ODMR spectra[3]. The ground state of an NV center is a spin triplet state ($S = 1$) with a zero-field splitting $D \approx 2.87$ GHz between the $m_S = 0$ and $m_S = \pm 1$ states defined along the NV axis. The Hamiltonian of the NV center is written as[4]

$$H_i = D\left(\mathbf{NV}_i \cdot \hat{\mathbf{S}}\right)^2 + \gamma_e \mathbf{B}\cdot\hat{\mathbf{S}}, \tag{4}$$

in the presence of a magnetic field **B**, where $\hat{\mathbf{S}} = (\hat{S}_x, \hat{S}_y, \hat{S}_z)$ is the spin-1 operator and $\gamma_\mathrm{e} \approx 28\ \mathrm{MHz\ mT^{-1}}$ is the electron gyromagnetic ratio. By diagonalizing the Hamiltonian, the transition frequencies $f_i^{\pm}$, up to the second order perturbation, are determined as[3]

$$f_i^{\pm} = D + \frac{3\gamma_\mathrm{e}^2 B^2}{2D}\sin^2\psi_i \pm \gamma_\mathrm{e} B\cos\psi_i \sqrt{1 + \frac{\gamma_\mathrm{e}^2 B^2}{4D^2}\tan^2\psi_i \sin^2\psi_i}, \tag{5}$$

where $\psi_i = \cos^{-1}(\mathbf{NV}_i \cdot \mathbf{B}/B)$ is the angle between the NV axis and the external magnetic field $\mathbf{B}$. For a given magnetic field, the orientation of the $\mathbf{NV}_i$ (and hence that of the ND) is determined from the angles ψ_i. However, if the ND is rotated by any angle about the magnetic field $\mathbf{B}$, the ODMR frequencies would not be changed. This ambiguity can be removed by introducing another magnetic field $\mathbf{B}'$ along a different direction from $\mathbf{B}$. Then another set of angles ψ_i' relative to $\mathbf{B}'$ can be obtained, which enables the unambiguous determination of the orientation of the ND. By the least-square fitting of the respective transition frequencies $f_i^{\pm}$ (with $\mathbf{B}$) and $f_i'^{\pm}$ (with $\mathbf{B}'$), with the Euler angles (α, β, γ) and the zero-field splitting D as the fitting parameters, we obtained the orientation of the ND. Once the ND orientation is determined, the comparison between the orientations before and after the rotation gives the rotation data $(\theta_{\mathrm{r}}, \phi_{\mathrm{r}}, \chi)$.

In practice, instead of fitting the transition frequencies, we directly fitted the four ODMR spectra under magnetic field $\mathbf{B}$ or $\mathbf{B}'$ before and after the rotation to deduce the rotation of the ND. The normalized ODMR spectrum is written as[4]

$$S(f) = b - \sum_i C_i \left[\frac{\Delta f^2}{4\left(f-f_i^+\right)^2+\Delta f^2} + \frac{\Delta f^2}{4\left(f-f_i^-\right)^2+\Delta f^2}\right], \tag{6}$$

where b is the baseline, C_i represents the ODMR contrast of the NV centres along the ith direction and Δf is the linewidth (FWHM). In the least-square fitting, the initial orientation of the ND were firstly deduced from the two ODMR spectra before the rotation by employing the Euler angles (α, β, γ), the baselines, the contrasts, the FWHM, and the shift of the zero-field splitting ΔD (slightly varied among NDs) as the fitting parameters. Then, the rotation of the ND were determined by fitting the two ODMR spectra after the rotation with the rotation axis and angle $(\theta_{\mathrm{r}}, \phi_{\mathrm{r}}, \chi)$, the contrasts, and the baselines as fitting parameters. The Euler angles, the FWHM, and ΔD were fixed to be the results obtained by fitting the spectra before the rotation. It was found

that for each ND, the contrasts and the baselines obtained by fitting the data before and after the rotation were approximately identical. The slight variation of the parameters was probably due to the laser and the MW power fluctuations between the ODMR measurements.

(3) Reconstruction of deformation from ND rotation data

The surface of the material before indentation is assumed to be horizonal ($z = 0$) with the surface normal $\hat{\mathbf{z}} = (0, 0, 1)$ (Supplementary Fig. 2e). The deformed surface after the AFM indentation is denoted as $z(\boldsymbol{\rho})$, where $\boldsymbol{\rho}$ is a point on the *x*-*y* plane. The normal vector of the deformed surface is defined as $\mathbf{n} = (-g_x, -g_y, 1)$, where $\mathbf{g}(\boldsymbol{\rho}) = \left(g_x, g_y\right) = \boldsymbol{\nabla}_{\boldsymbol{\rho}} z(\boldsymbol{\rho})$ is the gradient field of the deformed surface. If is the ND docked at point $\boldsymbol{\rho}$ has neither translation nor rotation relative to the surface, the unit normal vector of the deformed surface can be derived from the rotation data of the ND $\mathbf{R}(\theta_{\mathrm{r}}, \phi_{\mathrm{r}}, \chi)$ from

$$\hat{\mathbf{n}}(\boldsymbol{\rho}) = \left(n_x, n_y, n_z\right) = \mathbf{R}(\theta_{\mathrm{r}}, \phi_{\mathrm{r}}, \chi)\, \hat{\mathbf{z}}. \tag{7}$$

In turn, the gradient field $\mathbf{g}(\boldsymbol{\rho})$ is $\mathbf{g}(\boldsymbol{\rho}) = \left(-\frac{\hat{n}_x}{\hat{n}_z}, -\frac{\hat{n}_y}{\hat{n}_z}\right)$. The deformed surface $z(\boldsymbol{\rho})$ was reconstructed from $\mathbf{g}(\boldsymbol{\rho}_n)$ at a discrete set of locations $\{\boldsymbol{\rho}_n\}$, by minimizing the global least-square cost function[5]

$$\epsilon[z(\boldsymbol{\rho})] = \textstyle\sum_n \left|\mathbf{g}(\boldsymbol{\rho}_n) - \boldsymbol{\nabla}_{\boldsymbol{\rho}} z(\boldsymbol{\rho}_n)\right|^2. \tag{8}$$

(4) Deformation and ODMR simulation

The axisymmetric deformation $z(\rho)$ of a homogenous material caused by the indentation of an AFM tip (cone-shaped with tip radius 100 nm and half-angle 12°) was numerically simulated with a linear elastic model (the Hertz-Sneddon model)[6,7]. The result is shown as the blue line in Fig. 1c in the main text. For an ND docked on the deformed surface, the corresponding rotation

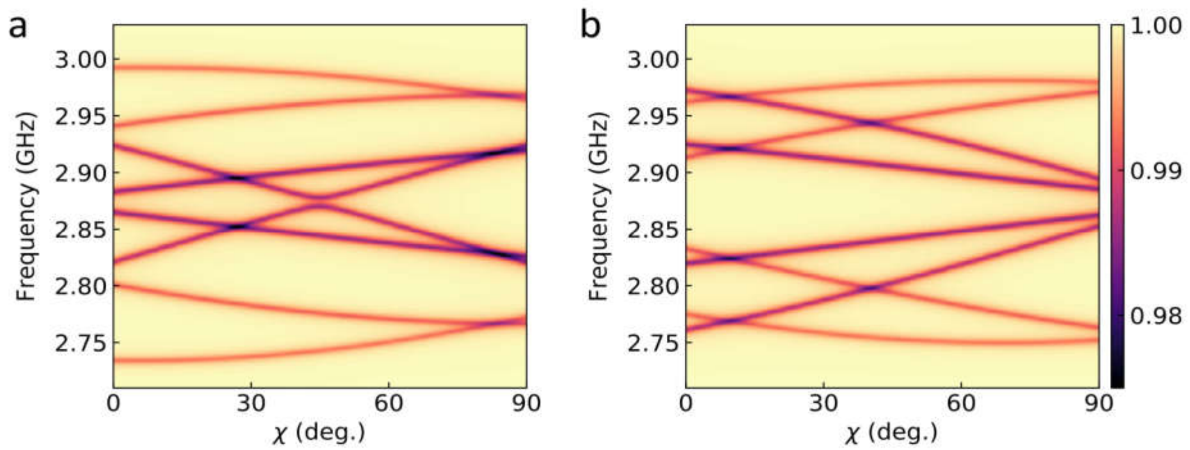

Supplementary Fig. 3 | Simulated optically detected magnetic resonance (ODMR) spectra as a function of the rotation angle χ under magnetic fields $\mathbf{B}$ (**a**) and $\mathbf{B}'$ (**b**).

angle χ of the ND induced by the indentation was calculated as $\chi(\rho) = \tan^{-1}[\partial_\rho z(\rho)]$ and the result is shown as the green line in Fig. 1c.

Taking an ND with initial orientation specified by the Euler angles $(\alpha, \beta, \gamma) = (45°, 20°, 65°)$ (configuration shown in Supplementary Fig. 2c) and rotated about the axis specified by $(\theta_\mathrm{r}, \phi_\mathrm{r}) = (90°, 25°)$, we simulated the ODMR spectra of the ND as a function of the rotation angle χ, for two different magnetic fields $\mathbf{B} = (60\ \mathrm{Gauss}, 5°, 90°)$ and $\mathbf{B}' = (60\ \mathrm{Gauss}, 30°, 270°)$ (shown in Supplementary Fig. 2c). The contrasts of the NV centers were set to $\{C_i\} = (0.7\%, 1.2\%, 0.8\%, 1.4\%)$ and $\Delta f = 7\ \mathrm{MHz}$ in all the cases. The contrasts and linewidths of the ODMR spectra with the two magnetic fields were set to be the same due to the small angle (35°) between the two fields. Two examples of such ODMR spectra (with the two rotation angles $\chi = 2°$ and 8°) are presented in Fig. 1c in the main text, and the frequency shifts in all simulated ODMR spectra are plotted in Supplementary Fig. 3.

Supplementary Note 3. Method validation

Feasibility of the proposal was first evaluated by measuring the rotation of a bulk diamond (containing NV ensembles) of known orientations. Figure S4a shows an optical image of the bulk diamond. We set the $(0\bar{1}1)$ and (011) axes of the diamond as the x- and y-axes, respectively. The four directions of the NV centers are shown in Supplementary Fig. 4b with color arrows. The corresponding Euler angles of this diamond were $(\alpha, \beta, \gamma) = (0°, 125.3°, 0°)$. The applied magnetic field **B** (light blue arrow in Supplementary Fig. 4b) had two components with one from a permanent magnet and the other from an electromagnet placed in the vicinity of the sample. $\mathbf{B}'$ (green arrow in Supplementary Fig. 4b) was obtained simply by inverting current flow direction in the electromagnet. Calibration of **B** and $\mathbf{B}'$ was carried out by fitting the ODMR spectra (black dotted lines in Supplementary Fig. 4c) taken from the bulk diamond (with the above specified Euler angles) under the magnetic field **B** or $\mathbf{B}'$. Least-square fitting was adopted (Supplementary Eq. 6), with the magnetic field, the shift of the zero-field splitting ΔD, the baselines b, the contrasts C_i and the linewidth Δf being the fitting parameters. The two magnetic fields were calibrated as $\mathbf{B} = (51.2\ \text{Gauss}, 84.2°, 169.7°)$ and $\mathbf{B}' = (54.5\ \text{Gauss}, 70.3°, 169.9°)$. The fitting results of the ODMR spectra are shown in grey lines in Supplementary Fig. 4c. The other fitting parameters were obtained as $\Delta D = 1.1$ MHz, $\{C_i\} = (1.6\%, 1.4\%, 1.1\%, 1.5\%)$, and $\Delta f = 6.4$ MHz (broadened by the ^{14}N hyperfine structure of the ODMR spectrum).

The bulk diamond sample was rotated counterclockwise about the z-axis. The optical image of the bulk diamond after the rotation is shown in the lower panel of Supplementary Fig. 4a. Two ODMR spectra under **B** or $\mathbf{B}'$ were acquired after the rotation (red dotted lines in Supplementary

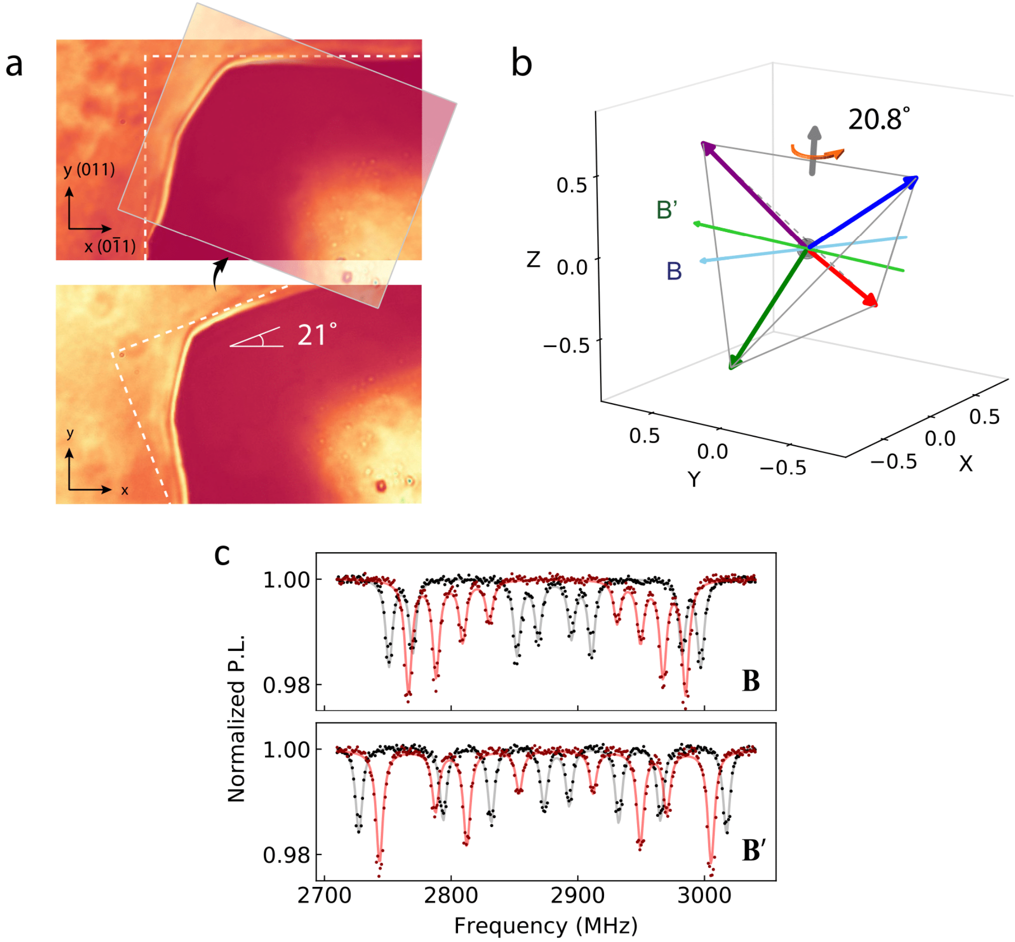

Supplementary Fig. 4 | Rotation measurement of a bulk diamond. **a,** Optical image of the bulk diamond before (upper) and after (bottom) the rotation. Before rotation, the sample was positioned with the $(0\bar{1}1)$ and (011) crystallographic axes along the *x*-, and *y*-axes of the laboratory frame, respectively. A 21° clockwise rotation is applied to the image after rotation in order to regain its original orientation. **b,** Schematic showing the configuration of the magnetic fields **B** and **B′**, and the orientations of the NV centers inside the bulk diamond. The grey arrow marks the rotation axis. **c,** Optically detected magnetic resonance (ODMR) spectra of the bulk diamond before (black dots) and after (red dots) rotation under the magnetic field **B** (upper) or **B′** (bottom). The grey and red lines are the best fitting.

Fig. 4c). By fitting the ODMR spectra with the known initial Euler angles of the diamond sample and the pre-calibrated magnetic fields, the rotation axis and angle were deduced as $(\theta_r, \phi_r, \chi) = (4°, 290°, 20.8°)$. The $\theta_r \sim 4°$ suggesting a small deviation of the rotation axis (grey arrow plotted in Supplementary Fig. 4b) from the *z*-axis ($\theta_r = 0°$). It was possibly caused by the manual manipulation of the sample stage for rotation operation. The deduced rotation angle $\chi \sim 20.8°$ was

consistent with the rotation angle ($\sim 21°$) measured from the optical images before and after the sample rotation (Supplementary Fig. 4a).

Supplementary Note 4. Reconstructing the deformation of a PDMS thin film

(1) Fabrication of PDMS films and their characterizations

PDMS was synthesized by mixing the precursor and cross linker with a ratio of 5:1. A microwave antenna was amounted a glass slide. The PDMS was then spin-coated onto the glass slide. After degassing, the surface of the PDMS film was modified by O_2 plasma that generated a surface oxidized layer[8,9]. At last, the NDs were spin coated on the PDMS film. The thickness of the PDMS film was measured to be ~ 50 μm (from optical measurement). To estimate the thickness of the oxidized surface layers, we prepared a cross-sectional PDMS sample by putting together two PDMS films with oxidized surfaces face to face. Figure S5a shows the AFM stiffness mapping of the cross-sectional PDMS. The dark region represents the bulk PDMS, and the bright region is the surface layer (with larger stiffness). A ~ 500 nm thickness of the surface oxidation layer was estimated from the thickness between the two dashed lines. AFM nanoindentation was performed on the bulk PDMS far from the oxidized surface, and apparent Young's modulus of the bulk PDMS was obtained as $E \sim 0.78$ MPa with the Hertz-Sneddon model[6,7].

A silicon nitride (with Young's modulus ~1 GPa) cantilever (DNP10-A Bruker) was applied to image and indent the PDMS film. After calibrating the spring constant and deflection sensitivity of this tip, the PDMS film was imaged in a peak force tapping mode and then indented in a ramp

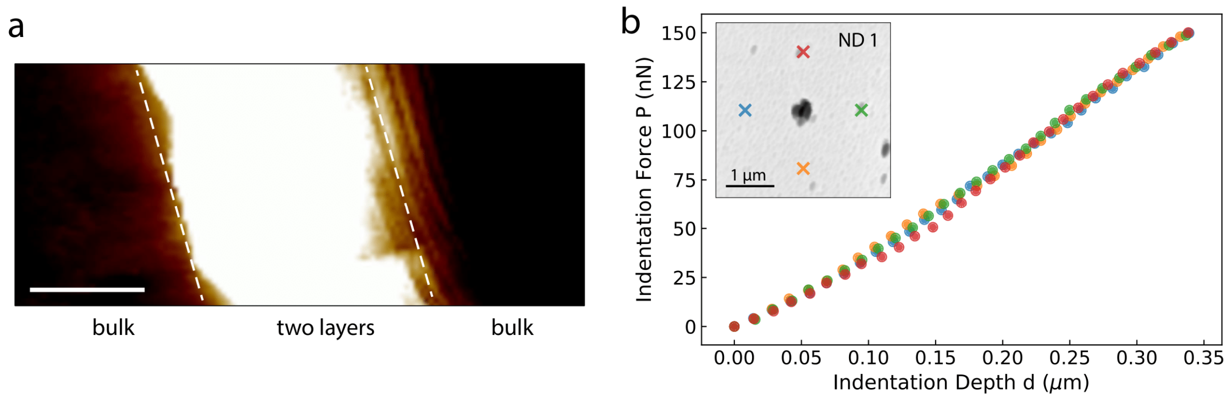

Supplementary Fig. 5 | Mechanical properties of oxidized polydimethylsiloxane (PDMS) film. **a,** The atomic force microscopy (AFM) stiffness mapping of a cross-sectional PDMS sample with surface oxidization. The white dashed lines separate the bulk and the surface layers. Scale bar is 500 nm. **b,** Force-depth profiles of the nanoindentations at different spots of the PDMS film. The inset shows the AFM image of the PDMS film, and the color crosses label the spots where the nanoindentations were applied.

mode. Figure S5b shows a few typical load-depth profiles obtained by nanoindentation performed at the spots labeled in the inset. The almost identical load-depth profiles of indentations carried out at different location on the sample suggest the homogeneity in the *x*-*y* plane of the PDMS film.

(2) Methods of orientation measurements of surface anchored NDs under an AFM tip indentation

In this protocol, dilute ND solution (2 μg mL^{-1}) was employed to avoid NDs aggregation. Dynamic light scattering (DLS) measurement of the ND solution suggested an average hydrodynamic diameter of 150 nm (Supplementary Fig. 6a). We also checked the sample using transmission electron microscopy (TEM) (Supplementary Fig. 6b), when reasonably dispersed NDs were found. The diameters of ND were in the range of a few tens of nanometers to ~200 nm, consistent with the DLS measurement. NDs were dispersed on the surface of the PDMS film by spin coating. Figure S6c shows the AFM topography image with NDs located on a 40 × 40 μm PDMS surface, correlated with the confocal image (Supplementary Fig. 6d) taken from the same

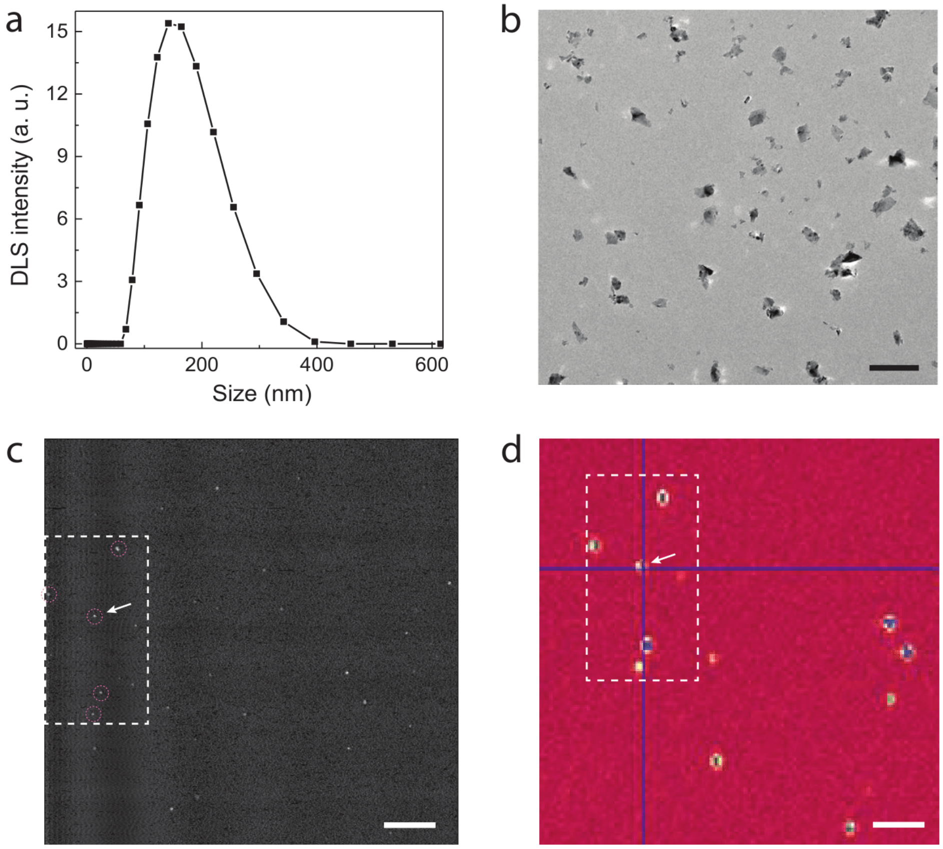

Supplementary Fig. 6 | Size distribution of nanodiamonds (NDs) and correlated atomic force microscopy (AFM) and fluorescence images. **a,** Size distribution of NDs measured by dynamical light scattering (DLS). **b,** Typical Transmission electron microscopy (TEM) image of NDs. Scale bar is 500 nm. **c,** AFM topography image with NDs (white dots) on a 40 × 40 μm polydimethylsiloxane (PDMS) surface. Some NDs are circled by pink dash lines. **d,** Confocal image taken from roughly the same region. The two images are correlated (marked by white dash rectangles) by the pattern of the NDs distribution. The white arrows indicate the same ND in the two images. Scale bars in **c** and **d** are 5 μm.

area by the pattern of ND spatial distribution (marked by the white dash rectangles). A DNP10-A AFM tip was applied for AFM imaging and nanoindentation experiments with calibrated spring constant of $0.39\ \mathrm{N\ m^{-1}}$ and deflection sensitivity of $42.28\ \mathrm{nm\ V^{-1}}$. The indentation experiment was carried out by lowing the AFM tip into the PDMS film in the proximity of a chosen ND (e.g. point 30 as shown in Fig. 2a in the main text) at a constant force $P = 150$ nN, with the indent and withdraw speed kept as 0.3 μm s^{-1} for total ramp size of 1 μm. ODMR spectra of the ND were recorded with and without an indentation under two pre-calibrated magnetic fields (using bulk

diamond, see Supplementary Note 3) $\mathbf{B} = (69.6\ \mathrm{Gauss}, 89.4°, 0.0°)$ and $\mathbf{B}' = (75.5\ \mathrm{Gauss}, 104.3°, 13.6°)$.

Using the methods described in Supplementary Note 2-2, the initial orientation of the ND (without indentation) was deduced from its ODMR spectra under $\mathbf{B}$ and $\mathbf{B}'$ and the corresponding fittings (black dots and lines in Supplementary Fig. 7). The characteristic Euler angles were determined as $(\alpha, \beta, \gamma) = (-156.2°, 85.1°, 71.2°)$. Rotation of the ND was then determined by fitting the ODMR spectra with the indentation as $(\theta_{\mathrm{r}}, \phi_{\mathrm{r}}, \chi) = (90°, 76°, 2.7°)$ (red dots and lines in Supplementary Fig. 7).

(3) Deformation reconstruction

For the *x-y* plane homogeneous medium, we adopted the single-ND method to reconstruct the deformation. Experimentally, the ND orientation sensor was fixed on a position with an AFM tip performing nanoindentation around the ND. This single-ND method is possible because on the homogeneous material the deformation at position A upon an indentation at position B should be the same as the deformation at B upon an indentation at A. The AFM indentation was performed in the proximity of the ND at the centre (0,0) in a programmed manner (indentation locations marked as red dots in Fig. 2a in the main text, the sequence of the indentations from 1-92 is marked by the blue arrows). The rotation of the ND upon each indentation was derived from the ODMR spectra for various displacements $\boldsymbol{\rho}$ (ρ, θ) of the ND from the AFM indentation positions (as shown in Fig. 2b in the main text). The deformation due to the indentation by an AFM tip applied to the origin (0,0) was reconstructed from the gradient field $\mathbf{g}(\boldsymbol{\rho})$ deduced by the rotation data of the ND (details see Supplementary Note 2-3). In the reconstruction process, the missing gradient

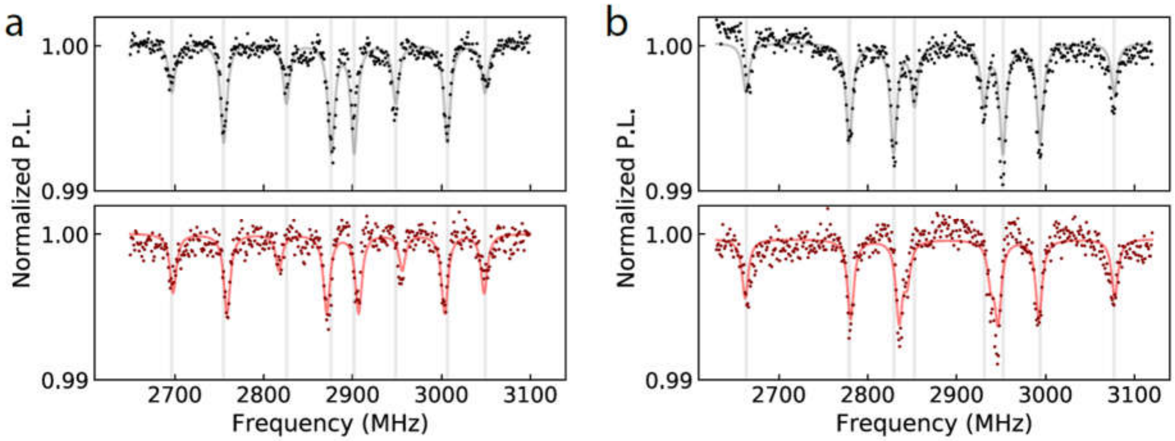

Supplementary Fig. 7 | Typical optically detected magnetic resonance (ODMR) spectra with and without an indentation. The ODMR spectra without (black dots) and with (red dots) indentation under magnetic field **B** (**a**) or **B′** (**b**). Black and red solid lines are the fitting results of the ODMR spectra. Grey lines indicate the positions of the resonance peaks before indentation.

field in the center and the edge of the deformed surface was estimated by the curvature-minimizing interpolation[10].

The experiments were repeated using different NDs at different locations of the PDMS film, and similar results were obtained. Here we show results obtained from other NDs as examples (ND2 and ND3). The AFM images of the two NDs are shown in Supplementary Fig. 8a and b. The same method as for ND 1 was adopted. The initial Euler angles of ND2 and ND3 were $(\alpha, \beta, \gamma) = (-5.0°, 112.7°, 21°)$ and $(-12.1°, 89.6°, -159.0°)$, respectively. By fitting the ODMR spectra, the rotations of the NDs with different indentations were obtained. Similar to data of ND1 presented in the main text (Fig. 2b), the rotations of ND2 and ND3 for various displacements $\boldsymbol{\rho}\,(\rho, \theta)$ of the NDs from the AFM indentation positions are shown in Supplementary Fig. 8c, and d, respectively, in which the arrows represent the projected rotation axes and the rotation angle. The rotation axis was always found on the tangential direction of the circle centered on the origin, which suggests that the NDs always rotated towards the indentation

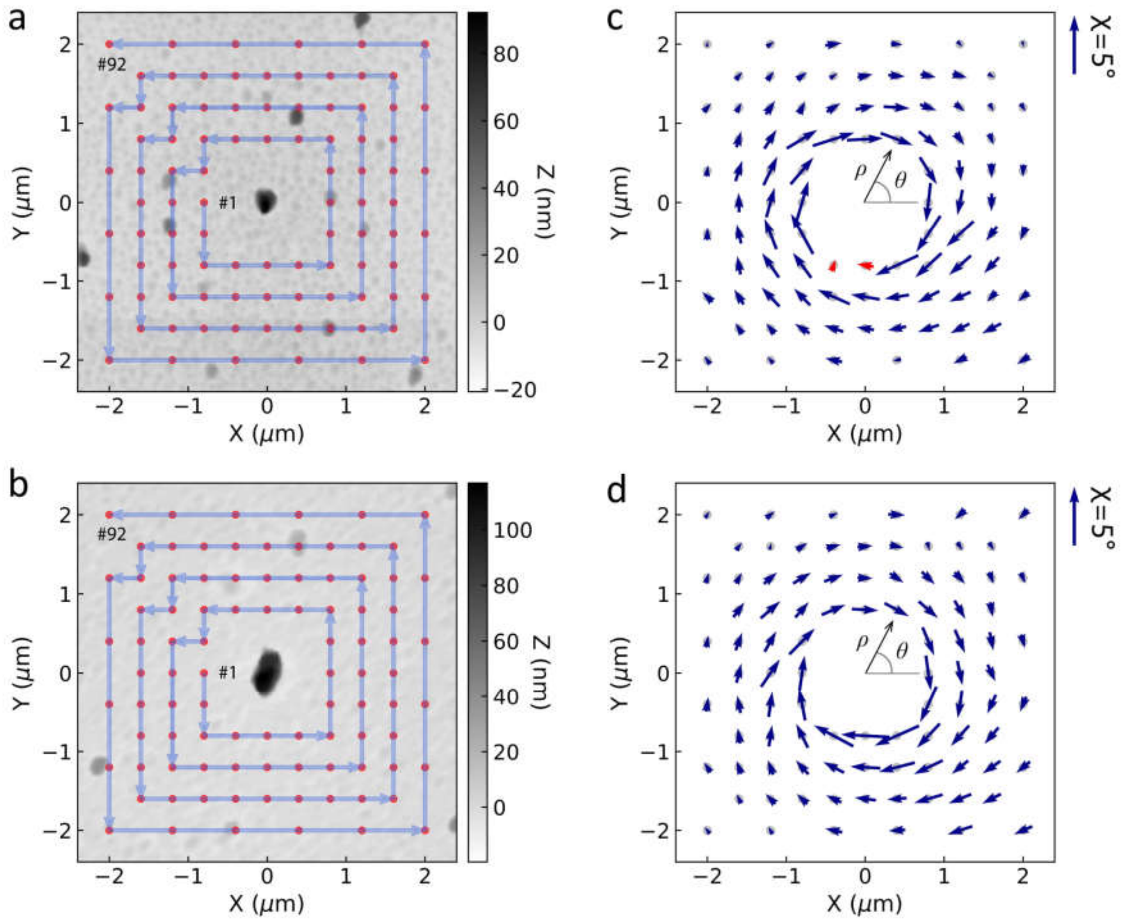

Supplementary Fig. 8 | Deformation reconstruction using the single-nanodiamond (ND) approach. **a** and **b,** Typical AFM images of the polydimethylsiloxane (PDMS) surface with the ND2 and ND3 located at the center (origin), respectively. The red dots represent the indentation locations of the atomic force microscopy (AFM) tip and the blue arrows indicate the indentation sequence, from #1 to #92. **c** and **d,** The rotation of the ND plot as arrows for the displacements of the ND2 and ND3, respectively, from the 92 indentation positions. The size of the arrows represents the magnitude of the rotation angles χ (scale bar shown on the right), and the direction is the rotation axes projected to the *x*-*y* plane.

point. The larger the separation distance between the indenting location and the NDs, the smaller the rotation angle. Two abnormal points in Supplementary Fig. 8c near the indentation points (marked by red arrows in Supplementary Fig. 8c) were likely to be induced by the tip-ND interactions when the indenting tip was very close to the ND — artificial displacement of NDs might have been induced. Together with results obtained from ND1 (presented in the main text), here we plot the polar angle θ_r and the azimuthal angle ϕ_r of the rotation axis for all three NDs as

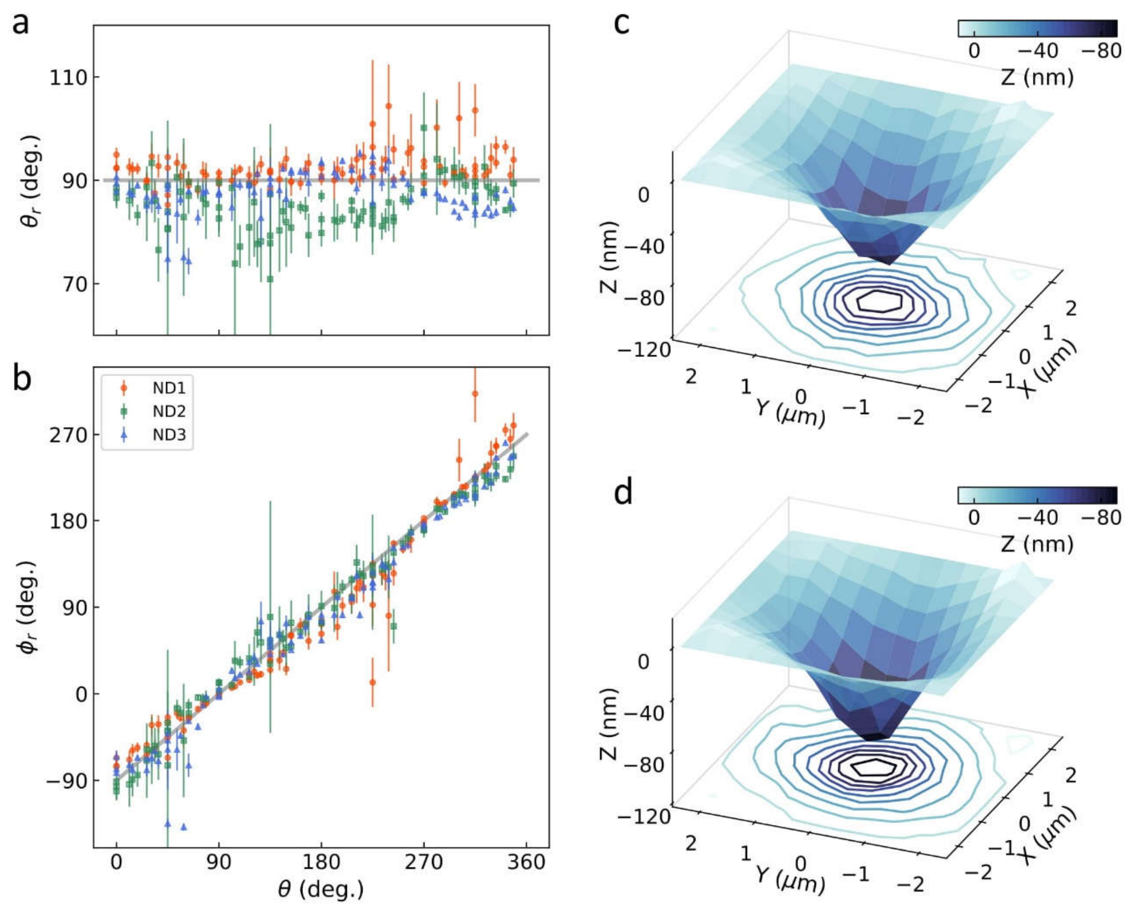

Supplementary Fig. 9 | Deformation reconstruction using the single-nanodiamond (ND) approach. **a,** The polar angle θ_r and **b,** the azimuthal angle ϕ_r of the rotation as functions of θ for the three NDs. The grey line in **b** is the condition $\phi_\mathrm{r} = \theta - 90°$. **c** and **d,** The reconstructed surface of the polydimethylsiloxane (PDMS) film during the atomic force microscopy (AFM) tip indentation for ND2 and ND3, respectively. The vertical error bars in **a** and **b** are the fitting errors.

functions of θ in Supplementary Fig. 9a and b, respectively. The rotation axes of all three NDs were approximately on the *x*-*y* plane (i.e., $\theta_\mathrm{r}{\sim}90°$) and were perpendicular to **ρ** (i.e., $\phi_\mathrm{r}{\sim}\theta - 90°$). The deformed surfaces of ND2 and 3 were reconstructed via the gradient fields as illustrated in Supplementary Fig. 9c, and d, respectively. The deformed surfaces were approximately axisymmetric about the AFM indentation at (0, 0).

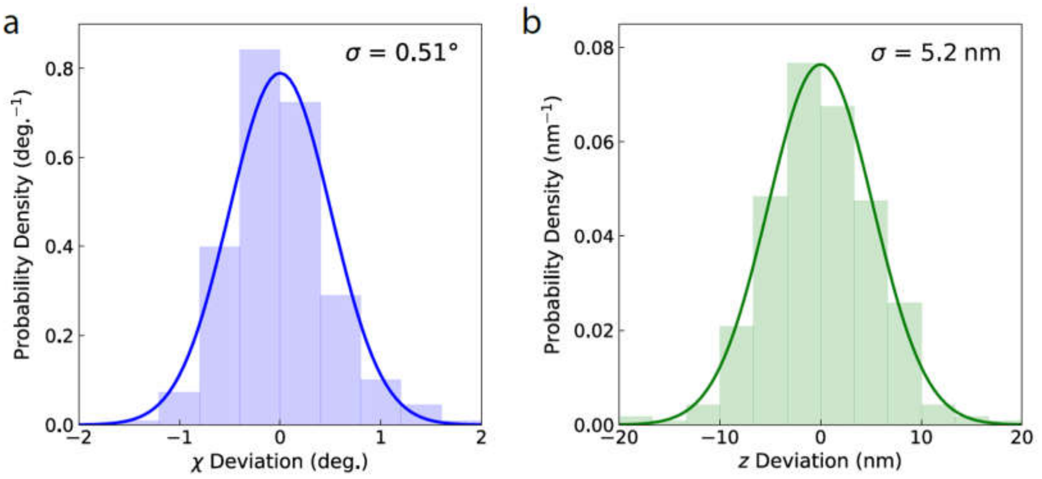

Supplementary Fig. 10 | The probability distribution of the deviation of **a,** the rotation angle χ and **b,** the z-direction deformation.

The deviations of the rotation angle (χ) and the reconstructed surface (z) measurement are defined as $\delta\chi = \chi - \langle\chi\rangle_\rho$ and $\delta z = z - \langle z\rangle_\rho$, where $\langle\chi\rangle_\rho$ and $\langle z\rangle_\rho$ are the respective mean value at the same ρ (distance between the ND and the AFM tip indentation) for each ND experiment. Probability distributions of the χ and z deviations for experimental data taken from all three NDs (blue and green shades) follow Gaussian distribution (blue and green lines) as illustrated in Supplementary Fig. 10a and b. The standard deviations for the rotation angle and reconstructed surface measurements were about $\sigma_\chi = 0.51°$ and $\sigma_z = 5.2$ nm, respectively. In the present experiments, a sufficiently long measurement time was ensured, so the precision of the deformation measurement was not shot-noise limited, but dominated by the environmental fluctuations, such as system mechanical vibration and temperature fluctuation.

AFM image and the ODMR spectra before and after the indentation experiments were compared for ND1 (Supplementary Fig. 11). Complete recovery of the PDMS surface after releasing the load is suggested by the absence of surface dent marks (Supplementary Fig. 11b). Identical patterns on the AFM images before and after the indentations (Supplementary Fig. 11a

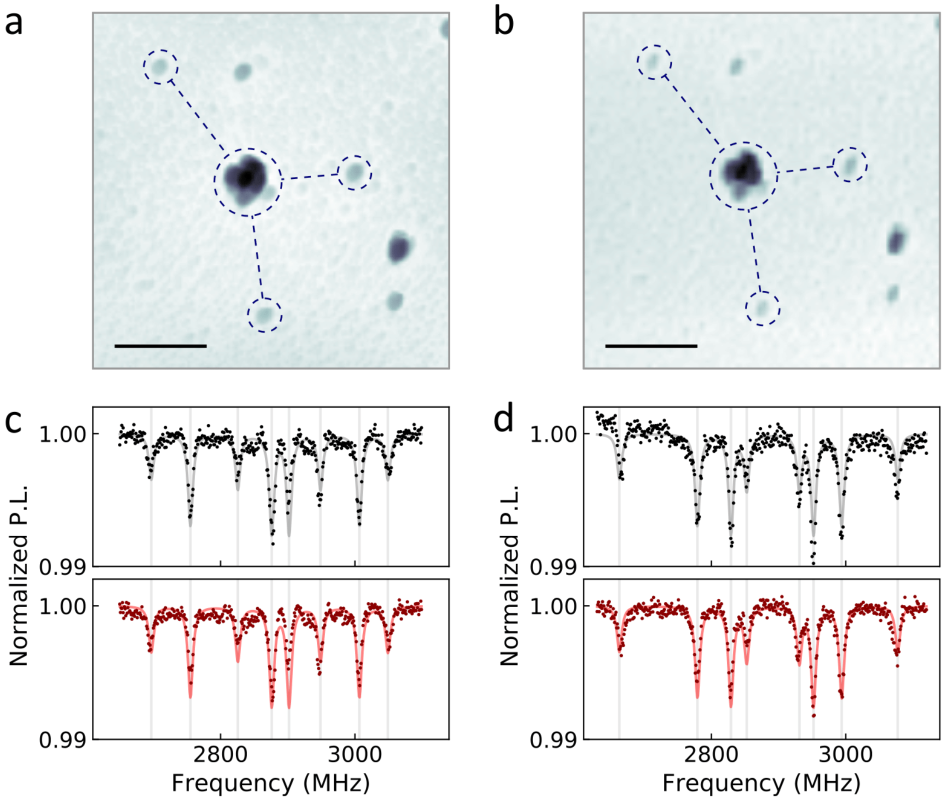

Supplementary Fig. 11 | The robustness of the measured nanodiamond (ND) in the experiments. **a** and **b,** The atomic force microscopy (AFM) image of the polydimethylsiloxane (PDMS) surface with the ND1 at center before, and after the indentation experiments, respectively. The patterns drawn in dashed lines matches each other in **a** and **b**. Scale bars are 1 μm. The optically detected magnetic resonence (ODMR) spectra before (black dots) and after (red dots) the indentations under magnetic field **B** (**c**) or **B**′ (**d**). Black and red lines are the fitting results of the ODMR spectra. Grey lines indicate the positions of the resonance peaks before indentation.

and b) indicate little relative motion of ND particles on the surface before and after the indentation experiments. Moreover, little change in the orientation of ND1 before and after the indentation was observed, as suggested by the identical resonance frequencies of the corresponding ODMR spectra (Supplementary Fig. 11c and d). These results suggest that the ND had little relative motion and rotation against the PDMS surface throughout the indentation experiments.

Supplementary Note 5. Simulation of the PDMS deformation.

The indentation induced deformation of PDMS film was simulated using a linear elastic model of a layer/bulk system under an axisymmetric loading, as illustrated in Supplementary Fig. 12a. The PDMS film (thickness ~50 μm) was modeled as a half-infinite substrate with a surface layer (formed due to oxygen plasma treatment as illustrated in Supplementary Note 4-1). Literature reports that the oxidized surface layer is stiffer than the bulk PDMS with approximately one-order of magnitude larger elastic modulus[9]. For simplicity, both the surface layer (with thickness t, Young's modulus E_1, and Poisson's ratio ν_1) and the bulk PDMS (Young's modulus E_0 and Poisson's ratio ν_0) were assumed to be isotropic. We adopted the same method as in Supplementary Ref. 11 to simulate the system. The parameters were chosen as $E_0 = 0.78$ MPa, $E_1 = 9.4$ MPa, $\nu_0 = \nu_1 = 0.33$, and $t = 500$ nm. The shape of the AFM tip was estimated as a cone with tip radius 50 nm and half-angle 25° (from SEM). The calculated indentation force-depth curve (grey line in Supplementary Fig. 12b) agrees well with the experiment results (blue

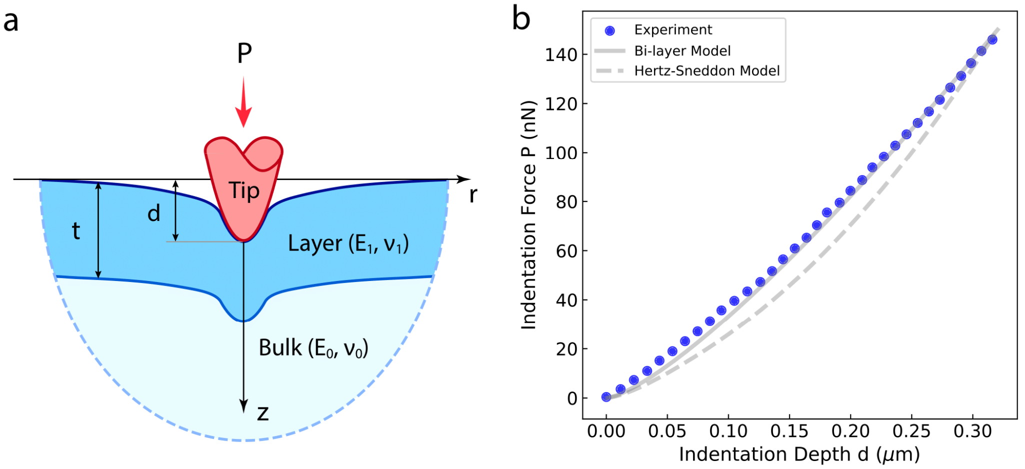

Supplementary Fig. 12 | Simulation of the polydimethylsiloxane (PDMS) deformation. **a,** Schematic of layer/bulk model under indentation. **b,** Indentation force-depth profile of the PDMS film (blue dots). Grey line is the simulation result for the layer/bulk model. Grey dash line is the result for Hertz-Sneddon model.

dots in Supplementary Fig. 12b) obtained by the AFM tip indentation on the PDMS sample. As a comparison, the dashed line shows the simulation force-depth profile obtained by the Hertz-Sneddon model[6,7], with the indented material assumed to be a half-infinity homogenous solid with an apparent Young's modulus of $E = 3$ MPa (Supplementary Fig. 12b).

Supplementary Note 6. Validation of specific contact models

The inadequate information obtained from the local deformation profile causes ambiguity in the choice of contact models to determine mechanical parameters, such as Young's modulus. In other word, more than one models may fit well the same local deformation profile. But they may lead to different non-local deformation responses. In the gelatin experiments (see Supplementary Note 7 and the main text), both the models with and without the effect of surface tension can be adopted to fit the local deformation profile (Supplementary Fig. 18), but only the model including the surface tension fits well the non-local deformation profile (Fig. 4 in the main text). The non-local deformation obtained by our method can be used to constrain different contact models.

As an additional example, the suitability of commonly used Johnson-Kendall-Roberts (JKR) model[12] and Derjaguin-Muller-Toporov (DMT) model[13] in analyzing the nanoindentation on adhesive materials can be investigated with our methods. The JKR model considers the effect of contact pressure and adhesion only inside the area of contact, while the DMT model assumes that the contact profile remains the same as in Hertzian model (without adhesion) but with additional attractive interactions outside the area of contact. We ran numerical simulations of the deformation upon nanoindentation using both JKR and DMT models[14], the two similar depth-loading curves

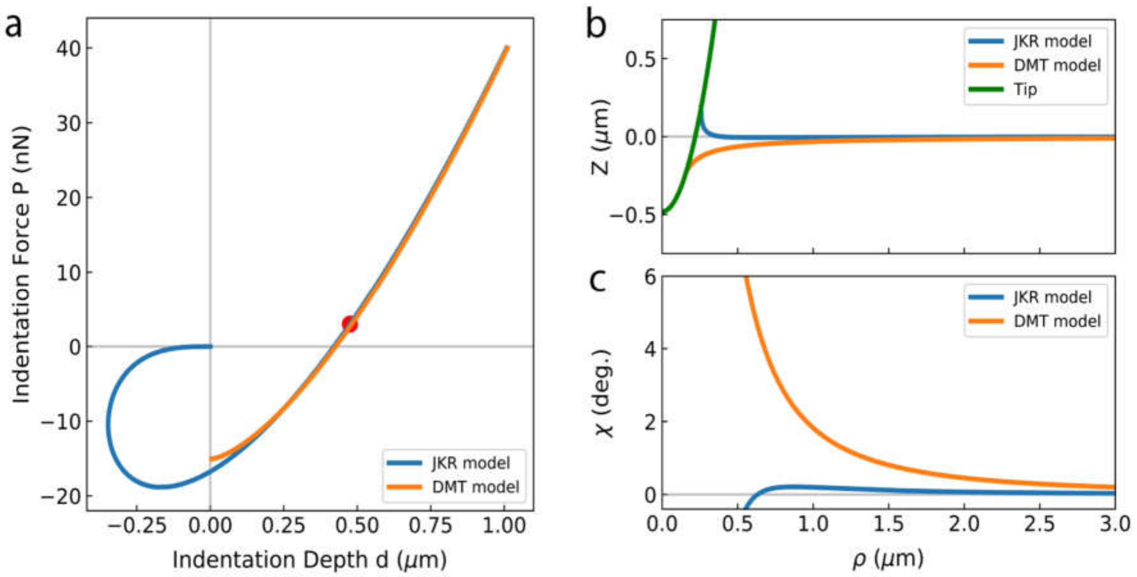

Supplementary Fig. 13 | Simulations for JKR and DMT models. **a.** Simulation results of the depth-loading curves based on JKR (blue) and DMT (orange) models. (b) and (c) Simulation results of the non-local deformations and the corresponding rotation angles as functions of the distance from the tip based on JKR (blue) and DMT (orange) models. The green line is the shape of the tip. The indentation force is set as 3 nN (red dot in (a)).

(Supplementary Fig. 13a) based on the two models gave different Young's modulus and adhesion energy ($E_{\mathrm{DMT}}/E_{\mathrm{JKR}} = 1.02$ and $W_{\mathrm{DMT}}/W_{\mathrm{JKR}} = 0.6$, where $E_{\mathrm{JKR}} = 80\ \mathrm{kPa}$ and $W_{\mathrm{JKR}} = 80\ \mathrm{mN\ m^{-1}}$). One would not be able to validate the choice of contact models and consequently determine the mechanical properties of samples based on only the local deformation profiles. On the contrary, when non-local deformations were considered, a significant difference was observed between the JKR and DMT models (Supplementary Fig. 13b), where the indentation force was chosen as $P = 3$ nN (indicated by the red spot in Supplementary Fig. 13a). The difference between the rotation angles of the surface ND (800 nm away from the indentation point) for the two models was ~3.3° (see Supplementary Fig. 13c), which was sufficiently large to be measured by the ODMR. Therefore, the indentation profile determination of homogeneous materials with high precision by our method provides additional information that can be used to validate the contact models.

Supplementary Note 7. Deformation reconstruction of gelatin upon AFM indentation.

(1) Fabrication and characterizations of the gelatin particles

In a standard procedure, 1.5 g gelatin was dissolved in 10 mL deionized water at 60 °C. At the same time, 0.2 g Span80 was dissolved in 40 mL toluene in a 100 mL round-bottom flask at 60 °C. Then 2 mL of the previously prepared $0.15\ \mathrm{g\ mL^{-1}}$ gelatin solution was added to the Span80 solution and they were uniformly mixed. After that, the gelatin in toluene emulsion was stirred at 300 rpm and cooled in ambient condition for 1 h. Then, the solidified particles were sonicated for 2 min. Finally, the particles were washed three times with 30 mL acetone, followed by another three times' wash using 30 mL deionized water/time.

We put the gelatin microparticles on the glass substrate and randomly dispersed the NDs on their top. To image and indent soft material in liquid environment, a silicon nitride cantilever

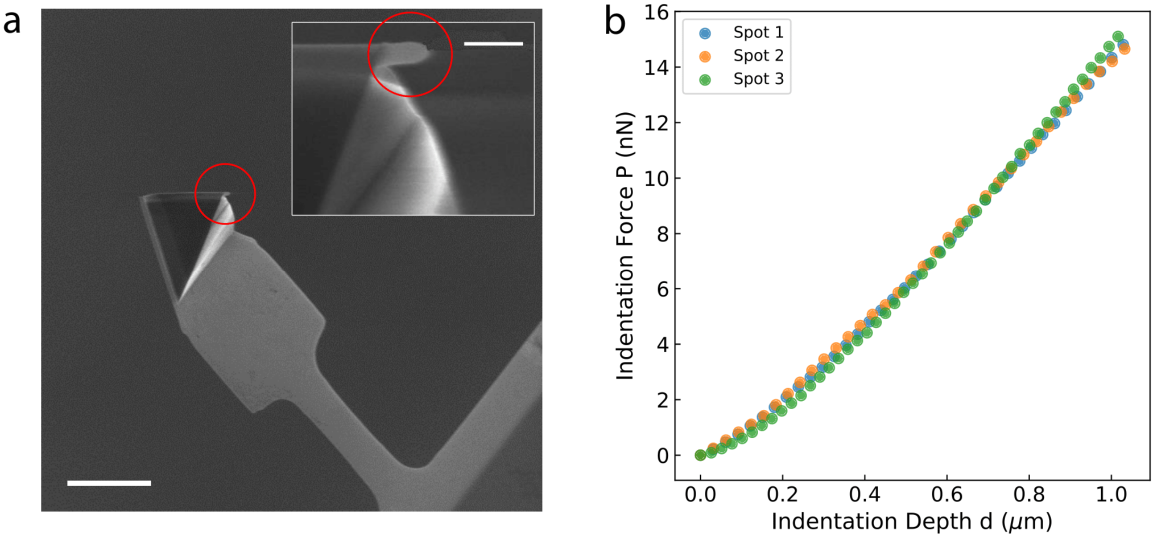

Supplementary Fig. 14 | Indentations on the gelatin particle. **a.** Scanning electron microscopy (SEM) images of a PFQNM-LC-A atomic force microscopy (AFM) cantilever used in the gelatin experiments. Scale bar is 10 μm and scale bar in inset is 1 μm. **b.** Force-depth profiles of the indentations at different spots on the gelatin particle.

(PFQNM-LC-A, Bruker) with small spring constant of $0.089\ \mathrm{N\ m^{-1}}$, deflection sensitivity of $21.01\ \mathrm{nm\ V^{-1}}$ and large tip radius of ~0.15 μm was used (as shown in Supplementary Fig. 14a). We imaged gelatin particles in peak force tapping mode with peak force of 300 pN (Fig. 4b in the main text). Indentations were applied on the gelatin samples at different locations using a PFQNM-LC-A cantilever in aqueous solution with holding force setpoint of 15 nN and indent and withdraw speed of $1\ \mathrm{\mu m\ s^{-1}}$ in total ramp size of 3 μm. Figure S14b shows the force-depth plots obtained by indentation performed at different location of the gelatin particle. Similar results were obtained, suggesting homogeneity in *x*-*y* plane of the gelatin particle.

(2) Orientation measurements of surface anchored NDs under an AFM indentation and deformation reconstruction

Deformation of gelatin was investigated using multiple NDs dispersed on the sample surfaces. Gelatin particles of 30 μm (diameter) were firstly dispersed on to a culture dish, and then 20 μL ND aqueous solution ($2\ \mathrm{\mu g\ mL^{-1}}$ concentration) was dropped on to the gelatin samples. After the NDs were docked on the gelatin samples, water was added to immerse both the sample and AFM tip. The indentation experiments were carried out in the center region of the gelatin particle, where the surface was close to a horizontal plane (the rms roughness was 20 nm with a height difference of ~ 150 nm from the center to the boundaries of the examined area; the separation distance from the center to the boundary was 2 μm). Upon any specific indentation, rotations of selected NDs at different distance away from the indentation location defines the gradient field there.

Eight sets of experiments were carried out on different locations on the gelatin (all within the central region to ensure that the starting planes are horizontal) with identical indentation force $P =$

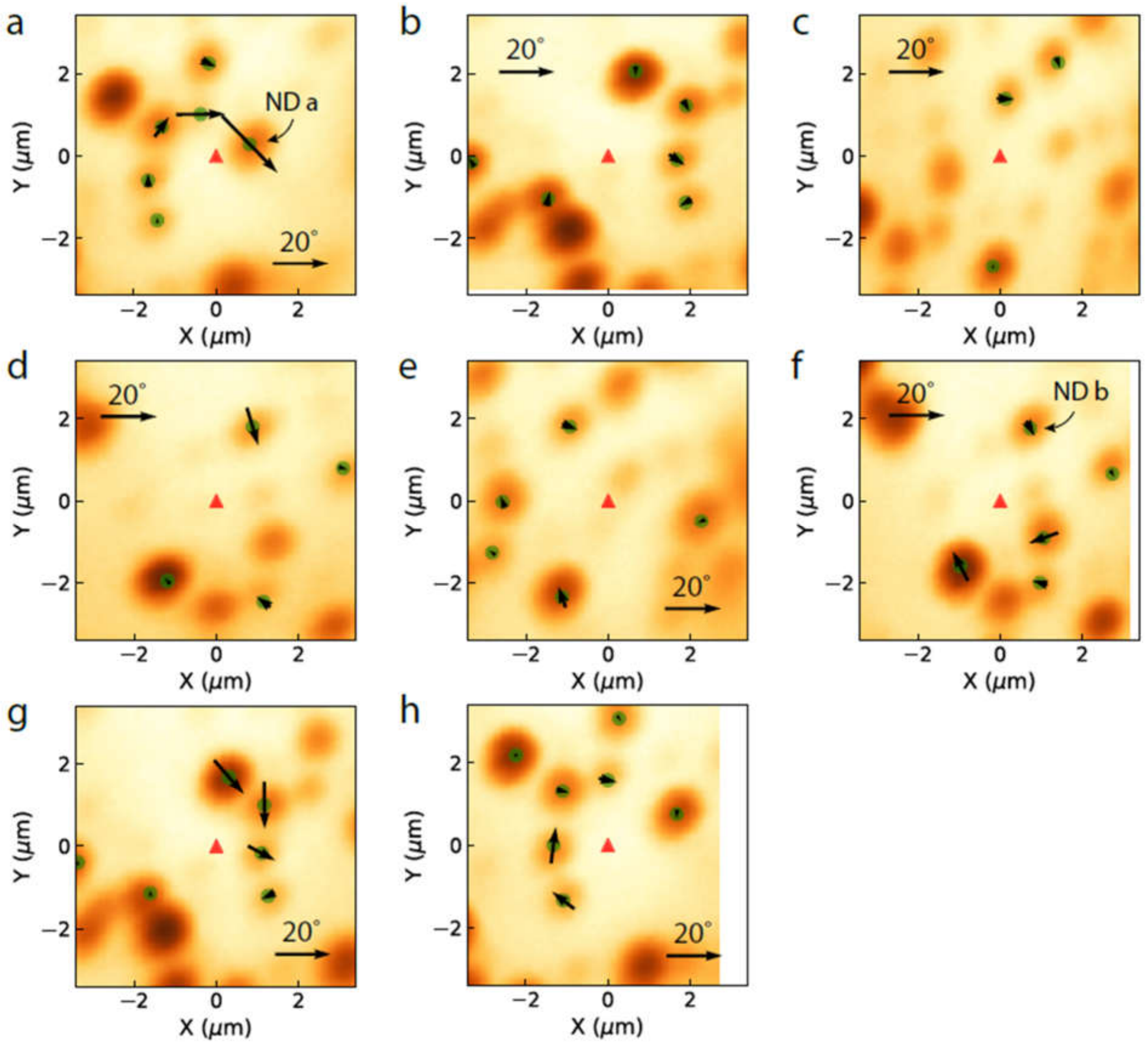

Supplementary Fig. 15 | Deformation reconstruction based on the multi-nanodiamond (ND) approach. **a to h,** Confocal images of gelatin with the indentation carried out at location 1 to 8 (red triangle in the figures). Green circles mark the positions of the NDs. The orientations and the sizes of the black arrows represent the direction of the rotation axes, and the magnitude of the rotation angles of the relevant NDs, respectively.

15 nN. The fluorescence images of these 8 sets of experiments are shown in Supplementary Fig. 15. The indentation location is marked by the red triangle, and the ND locations are marked by green circles. The distance between the indentation location and the measured NDs in each set of the experiments was determined by correlating the respective fluorescence image with the AFM image (for details, see Supplementary Note 1). Pre-calibrated magnetic field $\mathbf{B} =$

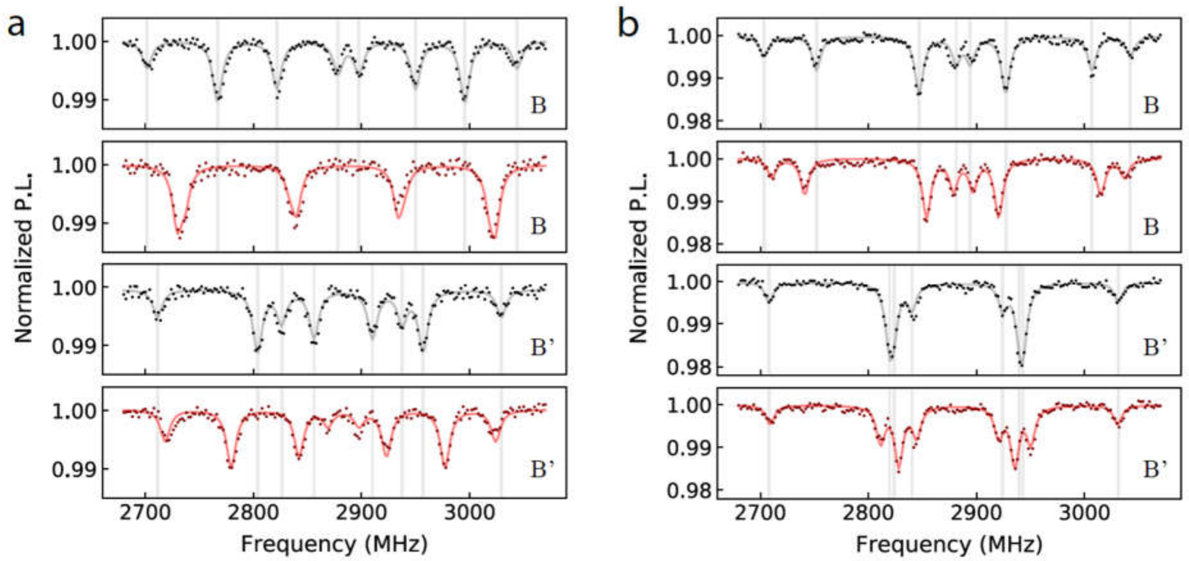

Supplementary Fig. 16 | Typical optically detected magnetic resonance (ODMR) spectra with and without indentations. The ODMR spectra without (black dots) and with (red dots) indentation under magnetic fields **B** (upper) or **B′** (bottom) taken from **a,** NDa (in experiment Set 1 as marked in Supplementary Fig. 15a) and **b,** NDb (in Set 6 as marked in Supplementary Fig. 15f). Black and red solid lines are the fitting results of the ODMR spectra. Grey lines indicate the positions of the resonance peaks before indentation.

$(66.7\ \text{Gauss}, 62.3°, 167.3°)$ or $\mathbf{B}' = (57.8\ \text{Gauss}, 84.8°, 167.3°)$ was applied. Figure S16 shows as examples the ODMR spectra taken from ND a (marked in Supplementary Fig. 15a) and b (marked in Supplementary Fig. 15f) with and without indentations. Without indentations, the characteristic Euler angles were determined as $(\alpha, \beta, \gamma)_{\mathrm{a}} = (156.4°, 83.6°, -60.4°)$ and $(\alpha, \beta, \gamma)_{\mathrm{b}} = (-16.9°, 93.2°, 172.2°)$ by fitting the ODMR spectra (black dots and lines in Supplementary Fig. 16). Rotations of the NDs were then determined by fitting the ODMR spectra with indentations, as $(\theta_{\mathrm{r}}, \phi_{\mathrm{r}}, \chi)_{\mathrm{a}} = (95.3°, 314.2°, 28.4°)$ and $(\theta_{\mathrm{r}}, \phi_{\mathrm{r}}, \chi)_{\mathrm{b}} = (110°, 295°, 5.7°)$ (red dots and lines in Supplementary Fig. 16). The deduced rotation axes and angles of the NDs in each of the indentation experiments are marked in the corresponding locations in Supplementary Fig. 15. Figure S17a and b plot the polar angles θ_{r} and the azimuthal angles ϕ_{r} of the rotation axis as functions of the polar angle θ (ND position in the polar coordinate (ρ, θ)), respectively. A

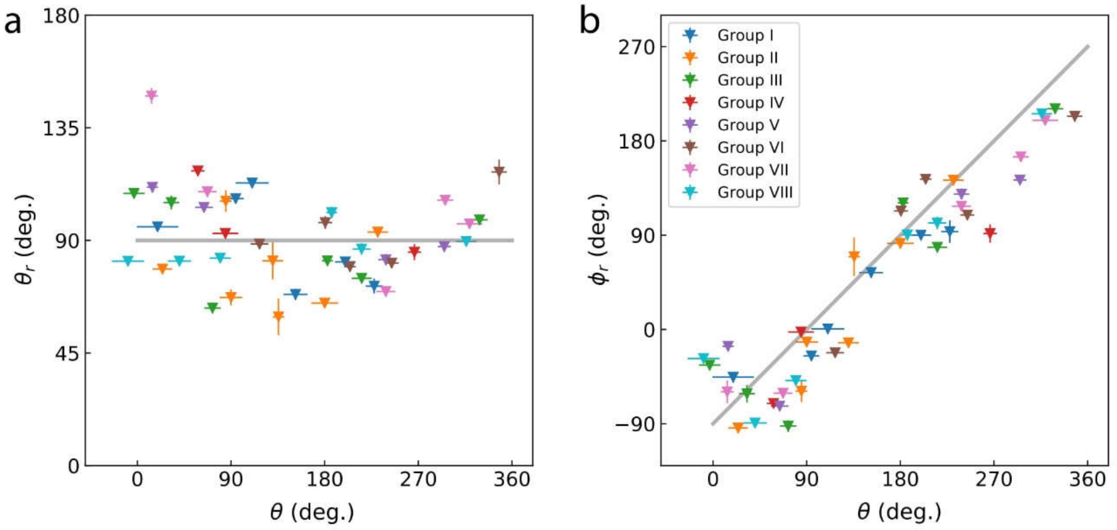

Supplementary Fig. 17 | The relation of the rotation axes and angles. **a,** The polar angle θ_{r} and **b,** the azimuthal angle ϕ_{r} of nanodiamonds (NDs) in the eight sets of experiments as functions of θ. The grey line in **b** is the axisymmetric condition $\phi_{\mathrm{r}} = \theta - 90°$. The horizontal and vertical error bars are the optical resolution and the fitting errors.

reasonable agreement exists between the experimental results and $\phi_{\mathrm{r}} = \theta - 90°$ (grey line in Supplementary Fig. 17b), suggesting that the NDs rotated toward the indentation location.

By assuming the axisymmetric condition, the gradient field can be written in polar coordinate as $(g_\rho, g_\theta) = (\tan\chi(\rho), 0)$. Then the deformed surface is reconstructed by integration, as $z(\rho) = \int_0^\rho \tan\chi(\rho')\, d\rho'$.

Supplementary Note 8. Simulation of the gelatin deformation.

Surface tension can play a major role in the mechanics of soft solids[15]. The dominance of surface tension or bulk elasticity can be evaluated using the elastocapillary length scale $L = \tau^0/E$, where τ^0 is the surface tension and E is the Young's modulus. When the scale of the deformation

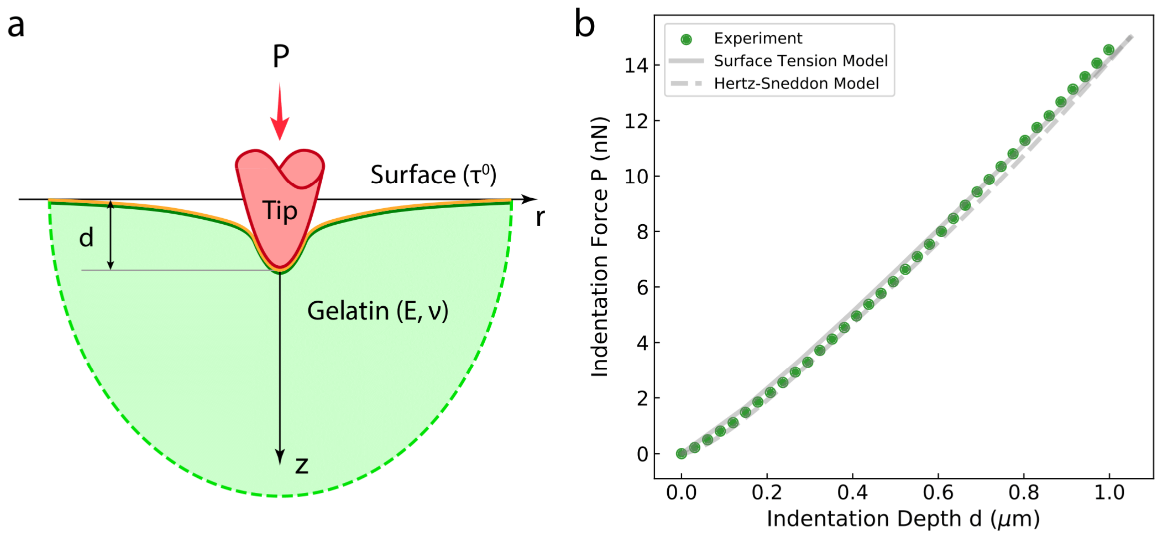

Supplementary Fig. 18 | Simulation of the gelatin deformation. **a,** Schematic of the system with surface tension upon indentation. **b,** The force-depth profile of the indentation on the gelatin (green dots). The grey solid line and dashed line represent the simulation results with and without the surface tension, respectively.

is much smaller than the elastocapillary length scale, surface tension dominates and flattens the surface. A linear-elastic model including the surface tension effect was employed to simulate the deformation of the gelatin upon AFM tip indentation (schematic shown in Supplementary Fig. 18a). In this model, the surface tension was introduced by adding a thin membrane of the same material ideally adhered to the bulk with negligibly thickness[16]. The system was simulated following the method in Supplementary Ref. 17. Young's modulus $E = 6.5$ kPa, Poisson's ratio $\nu = 0.5$ and surface tension $\tau^0 = 6.5$ mN m^{-1} were employed as the simulation parameters. The AFM tip was assumed a cone shape with tip radius 150 nm and half-angle 8° (according to the SEM imaging). The simulated indentation force-depth profile agrees with the experiment results (Supplementary Fig. 18b). The simulated result of force-depth profile using the Hertz-Sneddon model[6,7] (taking Young's modulus $E = 36$ kPa and zero surface tension $\tau^0 = 0$) is also plotted in Supplementary Fig. 18b (dashed line).

Supplementary Note 9. Sensitivity of the rotation measurement of the surface ND

We estimated the sensitivity of our sensing methods as follows. For optics based sensing, a fundamental limitation to sensitivity is the shot-noise of photon counts[4]. To estimate the sensitivity of our methods, we measured the rotations of the ND using various data acquisition time. Typical data was chosen as the ODMR spectra of the first indentation points around the ND3 on the PDMS film (see Supplementary Note 4). Figure S19 shows the measured standard deviation of the rotation angle σ_χ as a function of the data acquisition time t_m. The linear dependence of the standard deviations on the inverse square root of integration time gives the photon shot-noise-limited sensitivity. The sensitivities of the rotation angle measurement was deduced as $\eta_\chi \sim 1.1° \ \mathrm{Hz}^{-1/2}$.

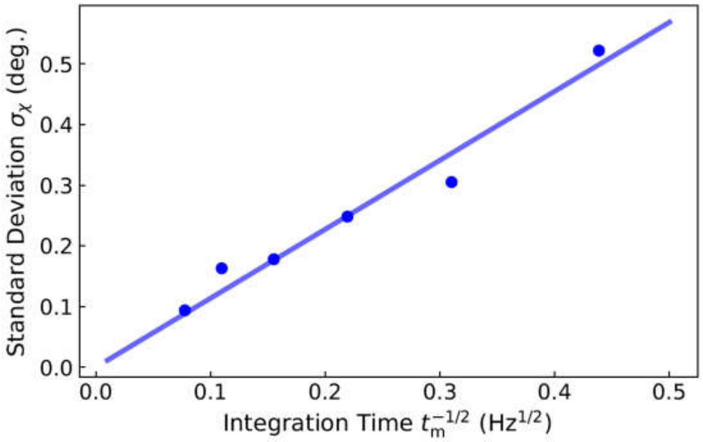

Supplementary Fig. 19 | The standard deviation of the rotation angle σ_χ as a function of the data acquisition time t_m. The line is the linear fitting with zero intercept.

Supplementary References:

1. Doherty, M. W. *et al.* The nitrogen-vacancy colour centre in diamond. *Phys. Rep.* **528**, 1–45 (2013).
2. Rose, M. E. *Elementary theory of angular momentum*. (Courier Corporation, 1957).
3. Doherty, M. W. *et al.* Measuring the defect structure orientation of a single NV − centre in

diamond. *New J. Phys.* **16**, 063067 (2014).

4. Rondin, L. *et al.* Magnetometry with nitrogen-vacancy defects in diamond. *Reports Prog. Phys.* **77**, 056503 (2014).
5. Harker, M. & O'Leary, P. Regularized Reconstruction of a Surface from its Measured Gradient Field. *J. Math. Imaging Vis.* **51**, 46–70 (2015).
6. Hertz, H. Ueber die Berührung fester elastischer Körper. *J. für die reine und Angew. Math. (Crelle's Journal)* **1882**, 156–171 (1882).
7. Sneddon, I. N. The relation between load and penetration in the axisymmetric boussinesq problem for a punch of arbitrary profile. *Int. J. Eng. Sci.* **3**, 47–57 (1965).
8. Bowden, N., Huck, W. T. S., Paul, K. E. & Whitesides, G. M. The controlled formation of ordered, sinusoidal structures by plasma oxidation of an elastomeric polymer. *Appl. Phys. Lett.* **75**, 2557–2559 (1999).
9. Mills, K. L., Zhu, X., Takayama, S. & Thouless, M. D. The mechanical properties of a surface-modified layer on polydimethylsiloxane. *J. Mater. Res.* **23**, 37–48 (2008).
10. Nielson, G. M. A Method for Interpolating Scattered Data Based Upon a Minimum Norm Network. *Math. Comput.* **40**, 253 (1983).
11. Li, J. & Chou, T.-W. Elastic field of a thin-film/substrate system under an axisymmetric loading. *Int. J. Solids Struct.* **34**, 4463–4478 (1997).
12. Johnson, K. L., Kendall, K. & Roberts, A. D. Surface Energy and the Contact of Elastic Solids. *Proc. R. Soc. A Math. Phys. Eng. Sci.* **324**, 301–313 (1971).
13. Derjaguin, B., Muller, V. & Toporov, Y. Effect of contact deformation on the adhesion of elastic solids. *J. Colloid. Interface Sci* **53**, 314–326 (1975).
14. Maugis, D. Adhesion of spheres: The JKR-DMT transition using a dugdale model. *J. Colloid Interface Sci.* **150**, 243–269 (1992).
15. Style, R. W., Jagota, A., Hui, C.-Y. & Dufresne, E. R. Elastocapillarity: Surface Tension and the Mechanics of Soft Solids. *Annu. Rev. Condens. Matter Phys.* **8**, 99–118 (2017).
16. Gurtin, M. E. & Ian Murdoch, A. A continuum theory of elastic material surfaces. *Arch. Ration. Mech. Anal.* **57**, 291–323 (1975).
17. Long, J. M. & Wang, G. F. Effects of surface tension on axisymmetric Hertzian contact problem. *Mech. Mater.* **56**, 65–70 (2013).
